# Highly luminescent InP/GaP/ZnS QDs emitting in the entire color range via a heating up process

**DOI:** 10.1038/srep30094

**Published:** 2016-07-20

**Authors:** Joong Pill Park, Jae-Joon Lee, Sang-Wook Kim

**Affiliations:** 1 Department of Molecular Science and Technology, Ajou University, Suwon 443-749, Korea; 2 Department of Energy & Materials Engineering, Dongguk University, Seoul 100-715, Korea

## Abstract

InP-based quantum dots (QDs) have attracted much attention for use in optical applications, and several types of QDs such as InP/ZnS, InP/ZnSeS, and InP/GaP/ZnS have been developed. However, early synthetic methods that involved rapid injection at high temperatures have not been able to reproducibly produce the required optical properties. They were also not able to support commercialization efforts successfully. Herein, we introduce a simple synthetic method for InP/GaP/ZnS core/shell/shell QDs via a heating process. The reaction was completed within 0.5 h and a full color range from blue to red was achieved. For emitting blue color, t-DDT was applied to prevent particle growth. From green to orange, color variation was achieved by adjusting the quantity of myristic acid. Utilizing large quantities of gallium chloride led to red color. With this method, we produced high-quality InP/GaP/ZnS QDs (blue QY: ~40%, FWHM: 50 nm; green QY: ~85%, FWHM: 41 nm; red QY: ~60%, FWHM: 65 nm). We utilized t-DDT as a new sulfur source. Compared with n-DDT, t-DDT was more reactive, which allowed for the formation of a thicker shell.

Quantum dots (QDs) exhibit very attractive characteristics as their properties including optical, electronic, and magnetic properties can be controlled by changing the particle sizes^1^,^2^,^3^. These materials have been intensively studied for several applications owing to their unique properties, especially in optical applications such as down conversion lighting^4^,^5^,^6^,^7^ and electroluminescence^8^,^9^,^10^. CdSe is one of the most studied QDs that exhibit outstanding optical properties^11^,^12^,^13^,^14^ including a narrow full width at half maximum (FWHM), high quantum yield (QY), and good stability. However, practical applications of CdSe are limited considering the environmental concerns owing to the cadmium toxicity. Several researchers have focused on reducing the toxicity of QDs as well as improving their optical properties. As an alternative, copper indium sulfide (CIS)^15^,^16^,^17^,^18^ is one of the materials exhibiting a high QY, but their ternary system cannot be used for covering the entire color range. In addition, they exhibit a low color purity and a narrow color gamut owing to their crystal imperfection. Recently, perovskites (CsPbX_3_, MAPbX_3_) QDs have received significant attention as an alternative material^19^,^20^,^21^,^22^,^23^,^24^,^25^. Perovskites exhibit an extremely narrow FWHM and a high QY over ~90%. Researches on perovskite QDs are still in the initial stage; further studies need to be carried out for exploring their applications. The issue of Pb toxicity should also be resolved. The most highlighted candidates for Cd-free QDs are the InP-based QDs^26^,^27^,^28^,^29^,^30^. However, synthetic conditions should be very carefully controlled because of the unstable precursors and the easily oxidizable products involved in the reaction. Therefore, progresses in the synthesis of InP-based QDs occurred much slowly compared to that of CdSe QDs. However, emergent demands owing to the environmental concerns and the practical applications of display devices spurred their development, resulting in a significant progress in a short period. Especially, the high QY and the narrow FWHM in the entire color range are significantly improved. On the contrary, the reliability in using these materials, which is associated with their thermal and chemical stabilities, needs further improvements.

In this work, we present efficient heating-up method for the synthesis of InP/GaP/ZnS QDs capable of reproducibly producing a full visible range (from blue to red) with great optical properties, including maximum QY and FWHM of 90% and 40 nm, respectively. As far as we know, these are the most improved results. In addition, the reaction time is very short as 15 min to 20 min. Based on our research, we expect that this process can be a stepping-stone towards the commercialization of InP based QDs.

## Results

**Figure 1:**
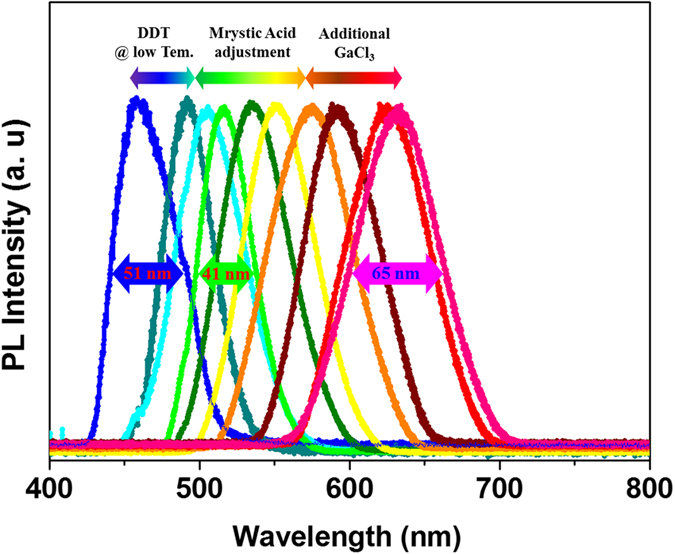
PL spectra of full visible region (1a). High quality InP based QDs are synthesized by easy and simple modification. Green sample shows the best results. (QY ~85%, FWHM 41nm).

InP/GaP/ZnS QDs which could cover full color ranges are synthesized in a very short time via a heating-up process. (Figure 1) Blue emitting QDs could be obtained by the addition of dodecanethiol (DDT) at low temperatures. The representative QY and FWHM is ~40% (max 50%), and 51 nm, respectively. Green to red emitting InP/GaP/ZnS QDs have been synthesized using a heating-up process by controlling the surfactant ratio and the reaction time. Green emitting QDs show the best performance. Representative QY is 85% (max 90%), and FWHM is very narrow. (41 nm) Red emitting dots show QY of ~60% (max 65%) and broad FWHM. (60 nm).

### Formation of core/inner shell structure

**Figure 2:**
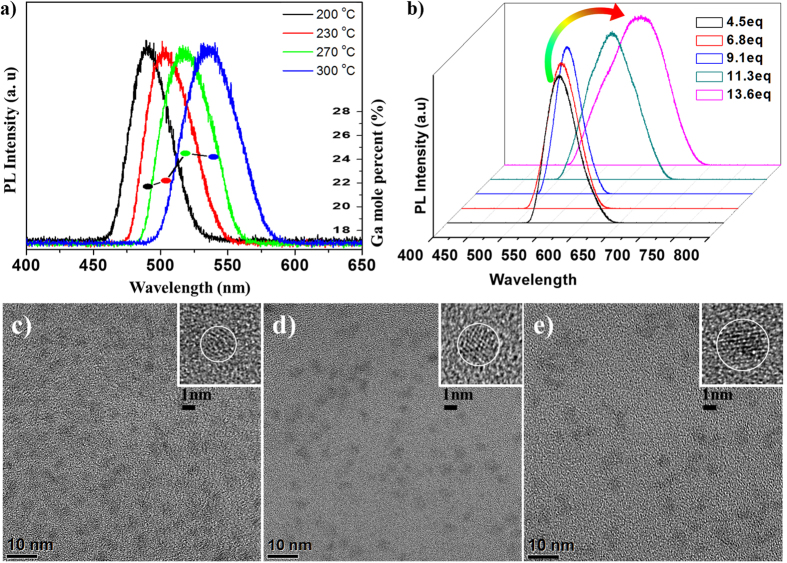
PL spectra change of InP/GaP core/inner shell growth. As the reaction temperature is elevated, spectrums move to a long wavelength region and contents of gallium are also increased. (1a) PL spectra of InP/GaP/ZnS QD. PL peaks are blue shifted as increase of surfactant (4.5 eq~9.1 eq). But excess amount of surfactant leads red shift and broadening of FWHM. (**b**) TEM images of three samples for different colors. Blue: 2.7 nm (**c**), Green: 3.2 nm (**d**), Red: 4.1 nm (**e**).

To prepare InP/GaP core/inner shell QDs using the heating up process, indium acetate (In(OAc)_3_) and zinc acetate (Zn(OAc)_2_) were added to octadecene containing myristic acid as a surfactant. Tris(trimethylsilyl)phosphine ((TMS)_3_P) was added to this mixture and the color of the solution changed to yellow^31^,^32^, which means the formation of indium-phosphine intermediate. Then, gallium chloride was added and the solution was heated to 300 °C. The color of the solution changed from yellow to wine red, indicating the formation of InP/GaP core/inner shell structures. For further characterization, InP/GaP core/shell QDs were collected after the general work-up process. In principle, this reaction can result in the formation of two different products, namely the InP/GaP core/shell and the In_x_Ga_1-x_P alloy structure, owing to their similar crystalline structures (zinc blende) and similar lattice parameters of GaP (a = 0.545 nm) and InP (a = 0.586 nm). If the reactivities of both the precursors are similar, the alloy structure can be obtained; otherwise, formation of the core/shell structure can occur. We examined the decomposition temperature of indium and gallium precursors under similar reaction conditions using the phosphine precursor, surfactant, and the solvent. Reaction of indium and phosphine occurred at comparatively low temperatures of 100 °C and Ga-P reaction occurred above 200 °C^33^. Therefore, we assume that the InP QDs were initially synthesized followed by coating of GaP on the InP core. In addition, correlation between the In/Ga elemental ratio and the emission wavelength could be used for examining the structure of the products. The alloy structure exhibits a blue-shifted emission with higher gallium content due to the wider bandgap of GaP compared with that of InP^34^. On the contrary, the InP/GaP core/shell structure exhibits a red-shifted emission with higher gallium content and thicker shells owing to the type-I band alignment. We obtained red emitting QDs with higher gallium content as compared with the green emitting QDs, thereby confirming the core/shell structure of the products. In a detailed experiment, aliquots of the reaction mixture were used for examining the emission property and stoichiometry at 200, 230, 270, and 300 °C and indicated a red shifted emission with high gallium contents at higher temperatures. (Figure 2a) A sample at 200 °C had a maximum emission peak at 470 nm, and then the emission peak shifted to 500, 520, and 545 nm as the temperature increased. A gallium content of 21% at 200 °C also changed to 21.5%, 24%, and 23.8% at each temperature (24% implies every precursor reacted quantitatively). The data indicated that the peak shift at 230 °C mainly resulted from the increased size of InP and that the gallium precursor was reactive above 230 °C. We suppose that the initial gallium content resulted from surface-attached gallium cations. To confirm the GaP shell, we synthesized InP/GaP shell which have thick GaP shell without ZnS and then observed that the peaks of PXRD move to the GaP reference. (Supporting Information. S2).

### Size control of core/inner shell

Recently, much research on InP QDs has been focused on the synthetic mechanism, which elucidated that the size of QDs depends on the reactivity of the intermediates in the reaction^35^,^36^,^37^. For example, Lewis bases such as oleylamine bind to indium of the In-P intermediate to compete with the phosphine precursors. The steric hindrance by oleylamine limits the growth process occurring between indium and (TMS)_3_P. We used DDT, which is a strong Lewis base for restricting the growth, thereby producing blue emitting small sized QDs. Green to orange color were obtained by controlling myristic acid content in the reaction, and the red color results from the use of increased gallium contents.

Surfactants, such as myristic acids, can be used for passivating the surface^38^ and adjusting the emission wavelength by controlling the particle size, as they not only bind to the precursors but also adsorb on the QD surface with a suitable strength, thereby decreasing the particle size as the surfactant concentration increases. In our experiment, myristic acid of 4.5 eq–13.6 eq of In(OAc)_3_ was used as large amounts of another cationic precursor Zn(OAc)_2_ were used. An emission wavelength of 570 nm (yellow color) is obtained with a myristic acid content of 4.5 eq, which shifts to 530 nm (green color) on increasing the surfactant amount to 9.1 eq. (Fig. 2b), also resulting in an improvement in the FWHM (50 nm). However, the addition of more amounts of surfactants (13.6 eq) has an opposite effect, causing a red shifted emission at 630 nm and a broad FWHM of 110 nm. These results indicate that surfactants with an optimum concentration can control the reaction between indium and phosphorus by steric hindrance^35^. Excess amounts of myristic acid lead to a strong inhibition of the reaction; nonetheless, the high reaction temperature of 300 °C invokes the reaction to produce red-shifted QDs exhibiting a broad FWHM. For producing good quality blue and red emitting QDs, the synthetic procedure should be modified. Then, we added thiol-based surfactants such as DDT in the middle of the reaction to prepare blue emitting QDs. The injection of DDT (1 mmol) was carried out at a relatively low temperature of 230 °C, as a result, ZnS outer shell was formed and the growth reaction was stopped. The emission wavelength can be tuned from 460 nm to 490 nm and the representative QY is 40% at 460 nm. High-quality red emitting QDs were synthesized by adding additional 30 mg of the gallium precursor (0.17 mmol). As stated above, gallium precursors form the GaP structure with TMS_3_P and induce red-shifted emission owing to the type-I band offsets. Higher amounts of gallium result in the formation of thicker inner shell and more red-shifted emissions. Formation of an additional ZnS outer shell results in a more red-shifted emission.

We measured the sizes of three samples (blue: 480 nm, green: 530 nm and red: 620 nm) by TEM. (Figure 2c) They were reacted with a same amount of DDT for thin shell formation. The sizes of particles are correlated with their colors. As it is well known, they show long wavelength emission as the particle size is bigger. The blue sample has the smallest size (2.7 nm), and the red sample has the biggest size (4.1 nm).

### Formation of ZnS outer shell

**Figure 3:**
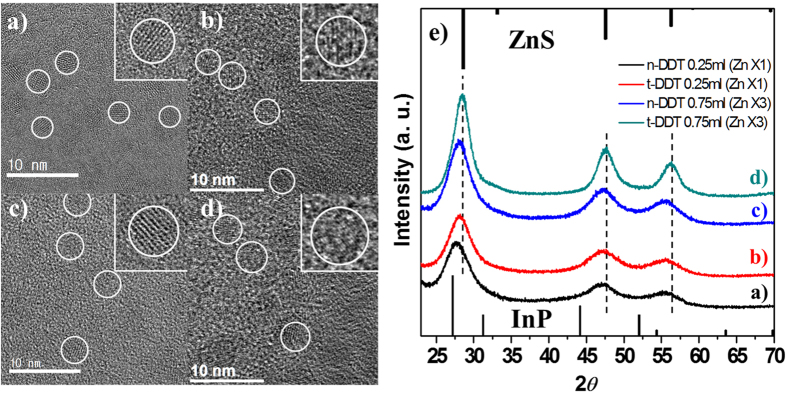
TEM images of QDs which used n-DDT (**a**: 0.25 ml, **c**: 0.75 ml) and t-DDT (**b**: 0.25 ml, **d**: 0.75 ml). Almost same sizes were obtained using 0.25 ml of t-DDT (**b**) and 0.75ml of n-DDT (**c**). Sample d shows the largest particles, and XRD pattern reveals thick ZnS shell (**e**).

ZnS has been widely used as an outer shell for various cores, such as CdE (E = S, Se, Te), InX (X = P, As), and CIS. These core/shell structures are favorable to QY because of their type-I band offsets owing to the wide bandgap of ZnS and the surface passivation. Several studies on the InP-based QDs/ZnS shell have been carried out; however, mismatched charge balance between InP (3^+^ and 3^−^) and ZnS (2^+^ and 2^−^) restrict the formation of a thick ZnS outer shell. As an alternative, insertion of a buffer layer (such as an In_2_O_3_ layer) was suggested. However, deteriorated emission properties limit the application of the produced materials^39^,^40^. In this work, tert-dodecanethiol (t-DDT) was used as the sulfur precursor for the ZnS shell. t-DDT is more reactive than n-DDT as tert-dodecane is more stable than n-dodecane, which are the products of the sulfur consuming reaction of t-DDT and n-DDT, respectively^41^,^42^. Therefore, a small amount of t-DDT is enough for producing a ZnS shell. In our work, the added DDT reacts with zinc precursor existing in the InP/GaP core solution, resulting in the production of a thin ZnS shell. X-ray diffraction (XRD) pattern in Fig. 3e indicates that the peak corresponding to (111) planes of QDs prepared using t-DDT is shifted more towards the ZnS direction. At this stage, the reaction cannot be proceeded further owing to the limited amounts of the zinc precursors used. Transmission electron microscopy (TEM) images (Fig. 3a–d) of the samples also indicate that bigger QDs of ~3.7 nm in diameter is produced by using t-DDT as compared to ~2.9 nm by using n-DDT. To form a thicker ZnS shell, a higher amount of zinc acetate and DDT are added to the solution and the reaction was carried out for 10 min. For comparison, equal amounts of n-DDT and t-DDT are used. XRD pattern in Fig. 3e indicates that the reaction using t-DDT is more progressed, similar to the previous reaction results. The XRD pattern shift toward the ZnS direction is an evidence of more ZnS shell. TEM images indicate clear changes in the shell thickness. Largest QDs of ~4.1 nm diameter are obtained using t-DDT. The samples exhibit similar optical properties such as the emission wavelength, QY, and FWHM even though different sized particles were obtained using different sulfur precursors.

The heat stabilities of the final products were investigated by aging them in a convection oven at 120 °C. The PL intensity change was observed over time. (Supplementary Information) They showed that the intensities of two different green emitted samples decreased only slightly (by ~5% in 24 h).

## Discussion

InP/GaP/ZnS QDs capable of reproducibly producing a full visible range (from blue to red) were developed using efficient heating-up process in a short reaction time of 0.5 hr. They showed excellent optical properties, including maximum QY and FWHM of 90% and 40 nm, respectively. Their optical properties and synthetic characteristics of heating up process allow mass production for display industry.

## Materials and Methods

All chemicals, indium acetate (In(OAc)_3_ 99.99% trace metal basis Sigma-Aldrich), zinc acetate (Zn(OAc)_2_ 99.99% trace metal basis Sigma-Aldrich), myristic acid (99% Sigma), oleic acid (90% technical grade Aldrich), gallium trichloride (GaCl_3_ beads, anhydrous 99.999% trace metal basis Aldrich), 1-octadecene (ODE 90% technical grade Aldrich), tris(trimethylsilyl)phosphine ((TMS)_3_P min 98% 10 wt% in hexane Strem, SK chemicals), were used without any further purification.

### Synthesis of InP/GaP/ZnS QD

#### Blue

Indium acetate (70 mg, 0.24 mmol), zinc acetate (183 mg, 1 mmol), and myristic acid (496 mg) were added to 4 ml of ODE in a 3-neck flask of 25 ml volume. The solution was degassed at 110 °C for 2 h under vacuum (solution A). Then, the solution was cooled down to room temperature (RT) and filled with N_2_ gas. (TMS)_3_P (480 mg, 0.19 mmol) was mixed with 1 ml of ODE in a glove box (solution B). GaCl_3_ beads (15 mg, 0.085 mmol) were added to 1 ml ODE and were dissolved under mild heating (solution C). The solutions B and C solutions were added to the solution A. Within 5 min, the color changed from white to yellow. The mixture was heated to 300 °C in 10 min. DDT (0.25 ml, 1 mmol) was added at ~200 °C–270 °C and the mixture was maintained for 10 min at 300 °C.

#### Green

Indium acetate (70 mg, 0.24 mmol), zinc acetate (183 mg, 1 mmol), and myristic acid (496 mg) were added to 4 ml of ODE in a 3-neck flask of 25 ml volume. The solution was degassed at 110 °C for 2 h under vacuum (solution A). Then, the solution was cooled down to room temperature (RT) and filled with N_2_ gas. (TMS)_3_P (480 mg, 0.19 mmol) was mixed with 1 ml of ODE in a glove box (solution B). GaCl_3_ beads (15 mg, 0.085 mmol) were added to 1 ml ODE and were dissolved under mild heating (solution C). The solutions B and C solutions were added to the solution A. Within 5 min, the color changed from white to yellow. The mixture was heated from RT to 300 °C within 10min, and growth for the target size. Finally, 0.25 ml of DDT was added and growth for 10 min. For the further growth of the shell, zinc oleate (2 mmol) was added before the DDT addition at 300 °C. DDT of 0.75 ml is added at once.

#### Red

Indium acetate (70 mg, 0.24 mmol), zinc acetate (183 mg, 1 mmol), and myristic acid (496 mg) were added to 4 ml of ODE in a 3-neck flask of 25 ml volume. The solution was degassed at 110 °C for 2 h under vacuum (solution A). Then, the solution was cooled down to room temperature (RT) and filled with N_2_ gas. (TMS)_3_P (480 mg, 0.19 mmol) was mixed with 1 ml of ODE in a glove box (solution B). GaCl_3_ beads (30 mg, 0.17 mmol) were added to 1 ml ODE and were dissolved under mild heating (solution C). The solutions B and C solutions were added to the solution A. Within 5 min, the color changed from white to yellow. The mixture was heated from RT to 300 °C within 10 min, and growth for the target size. Finally, 0.25 ml of DDT was added and growth for 10 min. For the further growth of the shell, zinc oleate (2 mmol) was added before the DDT addition at 300 °C. DDT of 0.75 ml is added at once.

For the further shell growth, zinc oleate (2 mmol) was added before the DDT addition at 300 °C. Finally, 0.75 ml of DDT was added and the mixture was maintained for 10 min.

### Characterization

TEM images were taken on a JEM-2100F (JEOL) operating at 200 kV. XRD patterns were obtained using a Rigaku Ultima III diffractometer equipped with a rotating anode and a Cu Kα radiation source (λ = 0.15418 nm). Inductively coupled plasma-optical emission spectrum (ICP-OES) was measured using OPTIMA 5300DV, PerkinElmer (US). Absorption spectra were measured by a Jasco V-670 UV/vis spectrophotometer. Photoluminescence (PL) emission spectra were obtained using USB 4000 (ocean optics) and FP-8100 (Jasco). Absolute PL quantum yield was measured by QE-1000 (Otsuka electronics), and was cross checked with organic dyes (Coumarin 153 (53%), Rhodamine 101 inner salt (91%) in ethanol). For the quantum yield measurements, OD was adjusted at 0.1.

## Additional Information

**How to cite this article**: Park, J. P. *et al*. Highly luminescent InP/GaP/ZnS QDs emitting in the entire color range via a heating up process. *Sci. Rep.* **6**, 30094; doi: 10.1038/srep30094 (2016).

## Supplementary Material

Supplementary Information
