# Supplementary material for: Highly luminescent InP/GaP/ZnS QDs emitting in the entire color range via a heating up process
Source: Sci Rep. 2016 Jul 20;6:30094. doi: 10.1038/srep30094 (PMC4951813; doi:10.1038/srep30094)
Supplement: Supplementary Information [file srep30094-s1.doc]

Highly luminescent InP/GaP/ZnS QDs emitting in the entire color range via a heating up process

*Joong Pill Park,^a^ Jae-Joon Lee^b^ and Sang-Wook Kim^a*^*

a Department of Molecular Science and Technology, Ajou University, Suwon 443-749, Korea.

b Department of Energy & Materials Engineering, Dongguk University Seoul 100-715, Korea

**
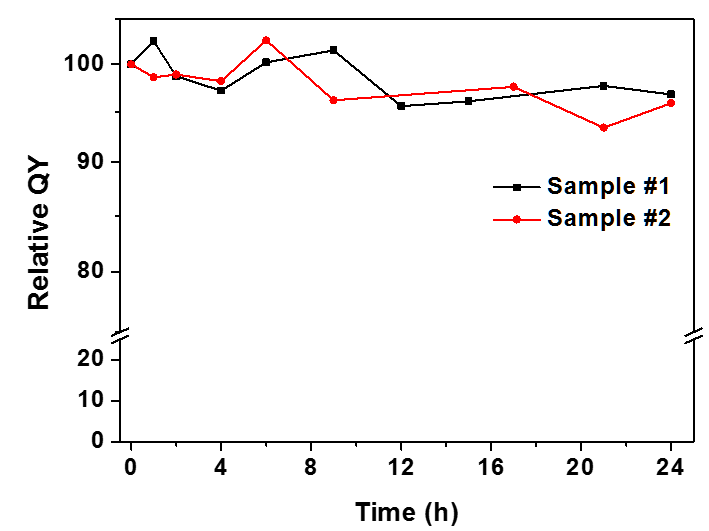
**

**S1.** Thermal stability data of two different InP/GaP/ZnS QDs. After 1day, the QY is still kept over 95%.


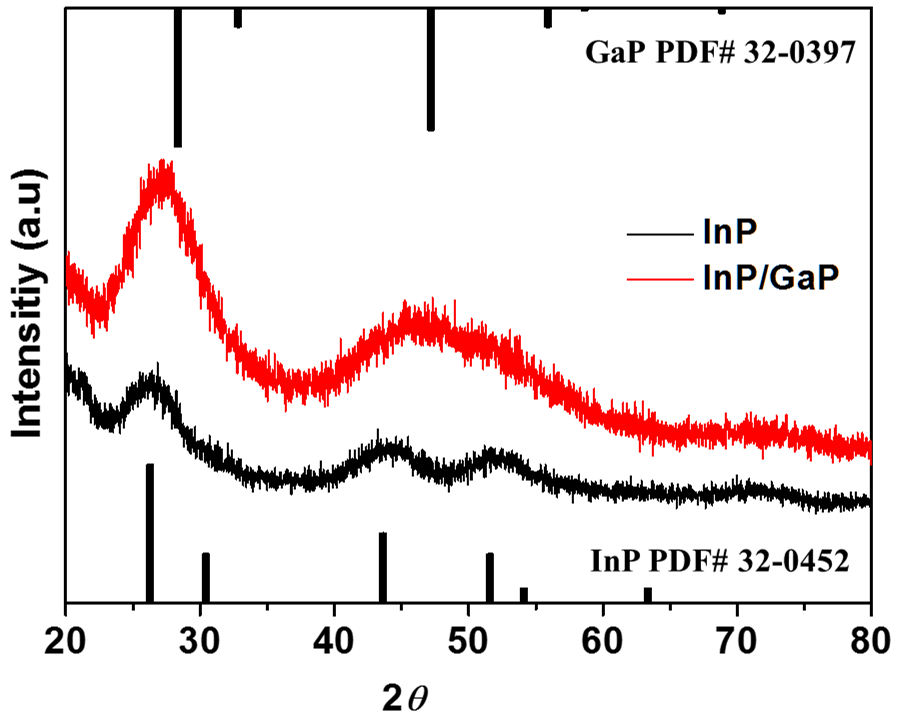


**S2.** PXRD data of InP/thick GaP shell.
